# C-reactive protein to albumin ratio as a predictor of early postoperative wound complications in patients undergoing posterior lumbar spine surgery

**DOI:** 10.3389/fsurg.2026.1842021

**Published:** 2026-05-28

**Authors:** Tao Niu, Haoran Zhang, Peng Xie, Shibin Shen, Haoran Huang, Zhenggang Zhou, Bin Wang, Jianlin Ma

**Affiliations:** 1 Department of Orthopedic Surgery, Qingdao Chengyang People’s Hospital, Qingdao, Shandong, China; 2 Department of Spinal Surgery, Peking University People’s Hospital, Peking University, Beijing, China; 3 Department of Orthopedic Surgery, People’s Hospital of Weiyuan, Dingxi, Gansu, China

**Keywords:** albumin, biomarker, complications, c-Reactive protein, spine surgery

## Abstract

**Objective:**

Early postoperative wound complications remain a significant challenge following posterior lumbar spine surgery. The C-reactive protein to albumin ratio (CAR) has emerged as a promising biomarker for systemic inflammation and nutritional status. This study aimed to evaluate the predictive value of preoperative CAR for postoperative wound complications in patients undergoing posterior lumbar spine surgery.

**Methods:**

We retrospectively reviewed 420 patients who underwent posterior lumbar spine surgery at our institution. Patients were divided into a case group (*N* = 140, with wound complications) and a control group (*N* = 280, without wound complications). Univariate and multivariate logistic regression analyses were performed to identify independent risk factors for early postoperative wound complications. Subgroup analyses were further conducted based on surgical strategy (interbody fusion vs. without interbody fusion) and surgical extent (number of levels >3 vs. ≤ 3).

**Results:**

Patients in the case group had significantly greater BMI, aCCI scores, ASA scores, blood loss, operative duration, and drain retention time compared to the control group (all *P* < 0.05). The proportions of diabetes mellitus and allogeneic blood transfusion were also significantly higher in the case group compared to controls (all *P* < 0.05). Notably, the proportion of patients with a high preoperative CAR (≥0.9) was significantly greater in the case group than in the control group (35.0% vs. 25.0%, *P* = 0.032). Multivariate logistic regression analysis revealed that a CAR ≥0.9 was an independent predictor of postoperative wound complications (fully adjusted OR: 1.753, 95% CI: 1.089–2.822, *P* = 0.021). In stratified analyses, CAR remained a robust predictor in patients undergoing interbody fusion (OR: 1.802, *P* = 0.011) and multilevel surgery (OR: 1.816, *P* = 0.010). However, CAR was not significantly associated with complications in patients undergoing surgery without interbody fusion (*P* = 0.726).

**Conclusion:**

Preoperative CAR is a valuable and independent biomarker for predicting early postoperative wound complications after posterior lumbar spine surgery. Its predictive utility is particularly significant in more complex procedures, such as interbody fusion and long-segment surgeries. Preoperative assessment of CAR may facilitate personalized risk stratification and clinical optimization to improve surgical outcomes.

## Introduction

Posterior lumbar spine surgery is currently one of the most widely performed interventions for the treatment of degenerative spinal pathologies, including lumbar stenosis, spondylolisthesis, and disc herniation (1). As the global population ages, the demand for these procedures to improve functional mobility and quality of life continues to rise. Despite significant advancements in surgical techniques, perioperative management, and aseptic protocols, early postoperative wound complications remain a prevalent and vexing challenge (2). These complications not only prolong hospital length of stay and increase healthcare costs but may also necessitate readmission or reoperation, leading to poor clinical outcomes and patient dissatisfaction (3–5).

Identifying patients at high risk for wound complications prior to surgery is crucial for risk stratification and the implementation of preventive strategies. Traditional risk factors such as advanced age, obesity, diabetes mellitus, and smoking are well-documented (6–8). However, relying solely on clinical history may not fully capture physiological and nutritional status. Consequently, there is growing interest in objective serum biomarkers that can reflect the systemic inflammatory and nutritional condition of the patient. C-reactive protein (CRP), a classic acute-phase reactant, and serum albumin, a marker of nutritional status, are routinely measured preoperatively. While valuable, interpreting them in isolation has limitations: CRP levels can fluctuate rapidly due to non-specific stimuli, and albumin levels can be influenced by hydration status and hepatic function (9, 10).

Recently, the C-reactive protein to albumin ratio (CAR) has emerged as a novel, composite inflammatory-nutritional biomarker (11). By combining the systemic inflammatory load (CRP) with the nutritional reserve (albumin), CAR provides a more stable and sensitive indicator of immune-inflammatory balance than either parameter alone (12). Previous studies have demonstrated the prognostic value of CAR in predicting outcomes in various malignancies and critical care settings (13, 14). In the field of orthopedics, elevated CAR has been associated with periprosthetic joint infection following arthroplasty and prognosis of spinal cord injury (15–17).

However, the specific relationship between preoperative CAR and postoperative aseptic wound complications in patients undergoing posterior lumbar spine surgery remains under-explored. Furthermore, the predictive value of CAR in the context of different surgical complexities, such as surgeries involving interbody fusion vs. posterolateral spinal fusion, or varying numbers of surgical levels, has not been fully elucidated. Therefore, the aim of this study was to evaluate the predictive value of preoperative CAR for early postoperative wound complications in patients undergoing posterior lumbar spine surgery. Additionally, we sought to determine if the predictive capability of CAR varies based on surgical strategy and the extent of surgery.

## Methods

### Study design and population

This is a single-center retrospective nested case-control study conducted at Peking University People’s Hospital. The study protocol was approved by the Institutional Review Board, and the requirement for informed consent was waived due to the retrospective nature of the analysis. We reviewed the medical records of patients who underwent posterior lumbar spine surgery between October 2015 and October 2024. Patients were consecutively enrolled. The spinal fusion types included posterior lumbar interbody fusion (PLIF) and posterolateral fusion (PLF).

The cohort was constructed first. The inclusion criteria were: (1) patients aged ≥ 18 years; (2) diagnosis of lumbar degenerative diseases (e.g., spinal stenosis, spondylolisthesis, disc herniation); and (3) availability of complete preoperative laboratory data, specifically CRP and albumin levels. Patients were excluded if they met any of the following criteria: (1) presence of active preoperative infection or inflammatory diseases (e.g., rheumatoid arthritis, ankylosing spondylitis); (2) history of previous spinal infection; (3) surgery for spinal tumors, trauma, or tuberculosis; or (4) incomplete medical records or lost to follow-up.

Patients experiencing early postoperative wound complications were selected from the cohort and included in the Case group. The date of wound complications diagnosis was defined as the index date. For each patient who developed wound complications, two control patients who had not experienced wound complications by the corresponding index date were selected from the same cohort. Controls were matched to cases by age, sex, and calendar year.

### Data collection

Demographic and perioperative data were extracted from the electronic medical record system. Preoperative variables included age, sex, body mass index (BMI), smoking status, diabetes status, age-adjusted Charlson Comorbidity Index (aCCI), and American Society of Anesthesiologists (ASA) physical status classification.

Surgical and perioperative details recorded included the duration of surgery, estimated blood loss, number of surgical levels, performance of interbody fusion (vs. PLF), use of bone grafting, drain placement, duration of drain retention, preoperative hospital stay, and requirement for allogeneic blood transfusion.

### Definition of CAR

Preoperative venous blood samples were collected prior to the surgical procedure. Serum levels of CRP (mg/L) and albumin (g/dL) were analyzed using standard laboratory techniques. The CAR was calculated using the formula: CAR = CRP (mg/L)/albumin (g/dL). To conduct statistical analysis, a cut-off value for CAR was established to categorize patients into high-CAR and low-CAR groups. Based on previous literature, a CAR value of 0.9 was utilized as the cut-off point (18).

### Outcome definition

The primary outcome of this study was the incidence of early postoperative wound complications. Early postoperative wound complications were defined as instances in which wound healing exceeds 2 weeks without the presence of surgical site infection. These complications include prolonged wound drainage (persistent drainage from the incision site persisting for more than 72 h postoperatively or requiring prolonged drain retention), hematoma, seroma, and wound dehiscence (separation of the wound edges requiring re-suturing or secondary healing) (19, 20). In our hospital, the criterion for drainage removal is a 24 h drainage volume of less than 100 mL.

### Statistical analysis

Statistical analyses were performed using SPSS software (Version 25.0, IBM Corp., Armonk, NY, USA). Continuous variables were tested for normality using the Kolmogorov–Smirnov test. Normally distributed data were expressed as mean ± standard deviation and compared using the Student t-test. Categorical variables were expressed as frequencies and percentages and compared using the Chi-square test.

To evaluate the independent predictive value of CAR for wound complications, univariate and multivariate logistic regression analyses were conducted. Results were reported as odds ratios (OR) with 95% confidence intervals (CI). Three regression models were constructed to control for confounding factors.

Furthermore, to determine if the predictive capability of CAR varied by surgical complexity, subgroup analyses were performed. The association between CAR and wound complications was stratified by surgical strategy (Interbody fusion vs. Without interbody fusion) and the extent of surgery (*N*umber of surgical levels >3 vs. ≤ 3). A *P* value of <0.05 was considered statistically significant.

## Results

### Baseline characteristics

A total of 420 patients were included in this study, comprising 140 patients in the case group and 280 patients in the control group. In the case group, 30 patients experienced prolonged wound drainage, 33 patients experienced hematoma, 12 patients experienced seroma, and 65 patients experienced wound dehiscence.

The demographic and clinical characteristics of the cohort are summarized in Table 1. There were no statistically significant differences between the two groups regarding age (*P* = 0.340), sex (*P* = 0.781), smoking status (*P* = 0.659), preoperative hospital stay (*P* = 0.111), use of bone grafting (*P* = 0.079), or drain placement (*P* = 0.688).

**Table 1:** General characteristics of the patient population.

Variables	Cases (*N* = 140)	Controls (*N* = 280)	*P* value
Age, years	62.7 ± 8.8	61.7 ± 10.7	0.340
Sex			0.781
Female	76 (54.3%)	156 (55.7%)	
Male	64 (45.7%)	124 (44.3%)	
Smoking status			0.659
Yes	28 (20.0%)	51 (18.2%)	
No	112 (80.0%)	229 (81.8%)	
Diabetes status			0.004*
Yes	46 (32.9%)	56 (20.0%)	
No	94 (67.1%)	224 (80.0%)	
BMI, kg/m2	27.7 ± 4.2	25.5 ± 3.6	<0.001*
aCCI	2.4 ± 1.2	1.5 ± 0.7	<0.001*
Preoperative hospital stay, days	2.7 ± 1.0	2.5 ± 1.3	0.111
Estimated blood loss, mL	276.2 ± 67.3	220.6 ± 59.3	<0.001*
Duration of surgery, min	193.7 ± 49.8	176.5 ± 54.9	0.002*
ASA score	1.7 ± 0.6	1.2 ± 0.4	<0.001*
Drain placement			0.688
Yes	129 (92.1%)	261 (93.2%)	
No	11 (7.9%)	19 (6.8%)	
Drain retention time, days	4.2 ± 1.1	3.2 ± 0.7	<0.001*
Allogeneic blood transfusion			0.013*
Yes	33 (23.6%)	39 (13.9%)	
No	107 (76.4%)	241 (86.1%)	
Bone grafting			0.079
Yes	123 (87.9%)	227 (81.1%)	
No	17 (12.1%)	53 (18.9%)	
Surgical types			0.352
PLIF	106 (75.7%)	200 (71.4%)	
PLF	34 (24.3%)	80 (28.6%)	
Number of surgical levels	3.6 ± 0.7	2.7 ± 0.8	<0.001*
CAR			0.032*
CAR < 0.9	91 (65.0%)	210 (75.0%)	
CAR ≥ 0.9	49 (35.0%)	70 (25.0%)	

BMI, body mass index; aCCI, age-adjusted charlson comorbidity index; ASA, American society of anesthesiologists; PLIF, posterior lumbar interbody fusion; PLF, posterolateral fusion; CAR, C-reactive protein to albumin ratio.

*P < 0.05

However, patients in the case group exhibited a significantly higher BMI compared to the control group (27.7 ± 4.2 kg/m^2^ vs. 25.5 ± 3.6 kg/m^2^, *P* < 0.001). The prevalence of diabetes mellitus was also significantly higher in the case group (32.9%) compared to controls (20.0%, *P* = 0.004). Additionally, patients with wound complications had higher aCCI scores (*P* < 0.001) and ASA scores (*P* < 0.001). Regarding perioperative variables, the case group was associated with greater estimated blood loss (*P* < 0.001), longer operative duration (*P* = 0.002), longer drain retention time (*P* < 0.001), and a higher rate of allogeneic blood transfusion (23.6% vs. 13.9%, *P* = 0.013). Notably, the proportion of patients with a high preoperative CAR (≥0.9) was significantly greater in the case group than in the control group (35.0% vs. 25.0%, *P* = 0.032).

### Association between CAR and early postoperative wound complications

Univariate and multivariate logistic regression analyses were performed to evaluate the predictive value of CAR for early postoperative wound complications (Table 2). In the crude model, a CAR ≥0.9 was associated with a 1.6-fold increase in the odds of wound complications (OR: 1.615, 95% CI: 1.040–2.509, *P* = 0.033). This association remained significant in the minimally adjusted model (Model 2), which controlled for BMI, aCCI, and ASA score (*P* = 0.029). After fully adjusting for potential confounders including diabetes status, estimated blood loss, duration of surgery, drain length, number of surgical levels, allogeneic blood transfusion, BMI, aCCI, and ASA score, elevated CAR (≥0.9) remained an independent risk factor for wound complications (OR: 1.753, 95% CI: 1.089–2.822, *P* = 0.021).

**Table 2:** Relationship between CAR and early postoperative wound complications. Values are odds ratio (OR) (95%CI).

Variable	Crude model^a^	Minimally adjusted model^b^	Fully adjusted model^c^
CAR			
CAR < 0.9	Ref	Ref	Ref
CAR ≥ 0.9	1.615 (1.040–2.509) *P* = 0.033*	1.652 (1.053–2.593) *P* = 0.029*	1.753 (1.089–2.822) *P* = 0.021*

BMI, body mass index; aCCI, age-adjusted Charlson comorbidity index; ASA, American Society of Anesthesiologists; CAR, C-reactive protein to albumin ratio.

a Crude model: we did not adjust other covariants.

b Minimally adjusted model: we adjusted BMI, aCCI and ASA score.

c Fully adjusted model: we adjusted diabetes status, estimated blood loss, duration of surgery, drain length, number of surgical levels, allogeneic blood transfusion, BMI, aCCI and ASA score.

*P < 0.05

### Stratified analysis by surgical strategy

To assess the robustness of CAR as a predictor across different surgical contexts, subgroup analyses were conducted based on surgical strategy (Table 3) and the number of surgical levels (Table 4).

**Table 3:** Relationship between CAR and early postoperative wound complications stratified by surgical strategy. Values are odds ratio (OR) (95%CI).

Variable	Crude model^a^	Minimally adjusted model^b^	Fully adjusted model^c^
Interbody fusion			
CAR < 0.9	Ref	Ref	Ref
CAR ≥ 0.9	1.745 (0.935–3.255) *P* = 0.080	1.757 (1.072–2.877) *P* = 0.025*	1.802 (1.147–2.831) *P* = 0.011*
Without interbody fusion			
CAR < 0.9	Ref	Ref	Ref
CAR ≥ 0.9	1.148 (0.897–1.468) *P* = 0.273	1.198 (0.620–2.316) *P* = 0.590	1.277 (0.325–5.014) *P* = 0.726

BMI, body mass index; aCCI, age-adjusted Charlson comorbidity index; ASA, American Society of Anesthesiologists; CAR, C-reactive protein to albumin ratio.

a Crude model: we did not adjust other covariants.

b Minimally adjusted model: we adjusted BMI, aCCI and ASA score.

c Fully adjusted model: we adjusted diabetes status, estimated blood loss, duration of surgery, drain length, number of surgical levels, allogeneic blood transfusion, BMI, aCCI and ASA score.

*P < 0.05

**Table 4:** Relationship between CAR and early postoperative wound complications stratified by number of surgical levels. Values are odds ratio (OR) (95%CI).

Variable	Crude model^a^	Minimally adjusted model^b^	Fully adjusted model^c^
Surgical levels > 3			
CAR < 0.9	Ref	Ref	Ref
CAR ≥ 0.9	1.746 (1.111–2.743) *P* = 0.016*	1.883 (0.898–3.950) *P* = 0.094	1.816 (1.154–2.857) *P* = 0.010*
Surgical levels ≤ 3			
CAR < 0.9	Ref	Ref	Ref
CAR ≥ 0.9	1.555 (0.955–2.533) *P* = 0.076	1.547 (1.000–2.392) *P* = 0.050*	1.667 (1.075–2.585) *P* = 0.023*

BMI, body mass index; aCCI, age-adjusted charlson comorbidity index; ASA, American society of anesthesiologists; CAR, C-reactive protein to albumin ratio.

a Crude model: we did not adjust other covariants.

b Minimally adjusted model: we adjusted BMI, aCCI and ASA score.

c Fully adjusted model: we adjusted diabetes status, estimated blood loss, duration of surgery, drain length, number of surgical levels, allogeneic blood transfusion, BMI, aCCI and ASA score.

*P < 0.05

When stratified by surgical strategy (Table 3), elevated CAR (≥0.9) was significantly associated with wound complications in patients undergoing interbody fusion in the fully adjusted model (OR: 1.802, 95% CI: 1.147–2.831, *P* = 0.011). In contrast, for patients undergoing posterior surgery without interbody fusion, CAR was not a significant predictor of wound complications in either the crude or adjusted models (*P* = 0.726 in the fully adjusted model).

When stratified by the number of surgical levels (Table 4), CAR demonstrated predictive value in both short- and long-segment surgeries. For patients undergoing multilevel surgery (>3 levels), CAR ≥0.9 was a significant independent predictor (OR: 1.816, 95% CI: 1.154–2.857, *P* = 0.010). Similarly, in patients with ≤3 surgical levels, the association remained significant in the fully adjusted model (OR: 1.667, 95% CI: 1.075–2.585, *P* = 0.023). These findings suggest that while high CAR is a risk factor regardless of the number of levels, the association is robust in both extensive and less extensive procedures, with a slightly higher OR observed in surgeries involving more than 3 levels.

## Discussion

The present study demonstrated that the preoperative CAR is a significant and independent predictor of early postoperative wound complications in patients undergoing posterior lumbar spine surgery. Even after adjusting for confounding variables, patients with a CAR ≥0.9 had a nearly 1.75-fold increased risk of developing wound complications. Importantly, our stratified analysis provides novel insights, revealing that the prognostic value of CAR is particularly robust in patients undergoing interbody fusion and in those undergoing multilevel surgery (>3 levels), highlighting the utility of CAR in risk-stratifying patients scheduled for complex spinal procedures.

Early identification of high-risk patients is paramount in spinal surgery to prevent catastrophic wound complications. While traditional markers like CRP and albumin have been widely used, their individual predictive power can be limited (21, 22). CRP levels can rise rapidly due to non-infectious stress, while albumin has a long half-life and may not reflect acute changes (23). CAR combines these two parameters into a single index, reflecting both the systemic inflammatory response and nutritional status. Consistent with findings in total joint arthroplasty and previous spinal infection studies (24, 25), our results confirm that an elevated CAR indicates a state of inflammatory-malnutrition, where the host immune response is overactive (high CRP) but the substrate for tissue repair is depleted (low albumin). This imbalance likely impairs collagen synthesis and fibroblast function at the incision site, thereby increasing susceptibility to hematoma formation, drainage, and infection (26).

A unique contribution of our study, distinguishing it from previous reports, is the stratified analysis based on surgical strategy. We found that CAR was a significant predictor for wound complications in the interbody fusion group but not in the non-interbody fusion group. Posterior lumbar interbody fusion (PLIF/TLIF) is inherently more invasive than simple decompression or posterolateral fusion alone, involving extensive soft tissue retraction, longer operative time, and the implantation of foreign cages (27). We hypothesize that in these high-demand surgical environments, the patient physiological reserve becomes the rate-limiting step for recovery. Patients with a high CAR lack the metabolic buffer to handle the substantial surgical stress of interbody fusion, leading to wound failure. In contrast, in less invasive non-interbody procedures, the surgical insult may be below the threshold where systemic inflammatory-nutritional status dictates the local wound outcome.

Furthermore, our analysis of surgical extent reinforces the robustness of CAR. We observed that CAR predicted complications in both short-segment and long-segment surgeries, with a slightly higher OR in the multilevel group. Multilevel surgery is associated with larger incision surface area, greater dead space, and increased blood loss (28). The fact that CAR remains predictive in short-segment surgeries suggests that even in routine cases, poor inflammatory-nutritional status can compromise outcomes. However, the stronger association in multilevel cases again supports the concept that as surgical complexity increases, the reliance on the patient intrinsic healing capacity (reflected by CAR) becomes more critical.

The findings of this study have practical implications for perioperative management. The calculation of CAR is simple, inexpensive, and relies on routinely available blood tests. We suggest that CAR should be incorporated into the preoperative screening protocol for posterior lumbar surgery. For patients with a CAR ≥0.9, especially those planned for interbody fusion, surgeons might consider prehabilitation strategies. This could include a short delay in surgery to optimize nutritional status through protein supplementation, or rigorous management of inflammatory comorbidities. Additionally, intraoperative preventive measures, such as the use of vancomycin powder or negative pressure wound therapy, might be more aggressively indicated for this high-risk subgroup.

There are several limitations to this study. First, as a retrospective analysis, the results may be subject to selection bias and may not be generalizable to other populations. Second, while we adjusted for multiple confounders, other factors such as the specific use of corticosteroids or the nutritional intake of patients were not controlled. Third, the definition of early postoperative wound complications is not adequately supported by the literature and is not widely adopted, which may hinder the application of the study findings to clinical practice. Furthermore, the definition includes some minor clinical events, which may exaggerate the incidence of complications. Finally, we grouped all wound complications together; a larger sample size would be needed to analyze specific complications (e.g., prolonged wound drainage vs. wound dehiscence) separately.

## Conclusions

In conclusion, preoperative CAR is a valuable, independent predictor of early postoperative wound complications in patients undergoing posterior lumbar spine surgery. Its predictive capability is most pronounced in patients undergoing interbody fusion and multilevel procedures. Assessing CAR preoperatively allows for better risk stratification and provides an opportunity for medical optimization to improve surgical safety and outcomes.

## Data Availability

The raw data supporting the conclusions of this article will be made available by the authors, without undue reservation.
